# Interference of Immunosuppressive Therapies with Cellular Antimicrobial Activity Against *Mycobacterium abscessus*

**DOI:** 10.3390/ijms27073230

**Published:** 2026-04-02

**Authors:** Sara Blanco-Conde, Miriam Retuerto-Guerrero, Ramiro López-Medrano, Cristina López-Cadenas, Santiago Vivas-Alegre, Elizabeth de Freitas-González, Nuria López-Morán, Octavio Miguel Rivero-Lezcano

**Affiliations:** 1 Servicio Análisis Clínicos, Complejo Hospitalario de Vigo, 36213 Vigo, Pontevedra, Spain; sl.blanconde@gmail.com; 2 Servicio de Reumatología, Complejo Asistencial Universitario de León, Gerencia Regional de Salud de Castilla y León (SACYL), Altos de Nava, s/n, 24071 León, Spain; mretuertog@saludcastillayleon.es; 3 Servicio de Microbiología Clínica, Complejo Asistencial Universitario de León, Gerencia Regional de Salud de Castilla y León (SACYL), Altos de Nava, s/n, 24071 León, Spain; rzlopez@saludcastillayleon.es; 4 Pharmacology, Department of Biomedical Sciences, Veterinary Faculty, Institute of Biomedicine (IBIOMED), University of León, 24071 León, Spain; 5 Servicio de Aparato Digestivo, Complejo Asistencial Universitario de León, Gerencia Regional de Salud de Castilla y León (SACYL), Altos de Nava, s/n, 24071 León, Spain; 6 Servicio de Neumología, Complejo Asistencial Universitario de León, Gerencia Regional de Salud de Castilla y León (SACYL), Altos de Nava, s/n, 24071 León, Spain; emfreitas@saludcastillayleon.es; 7 Laboratorio de Hematología, Complejo Asistencial Universitario de León, Gerencia Regional de Salud de Castilla y León (SACYL), Altos de Nava, s/n, 24071 León, Spain; 8 Unidad de Investigación, Complejo Asistencial Universitario de León, Gerencia Regional de Salud de Castilla y León (SACYL), Altos de Nava, s/n, 24071 León, Spain; 9 Institute of Biomedical Research of Salamanca (IBSAL), 37007 Salamanca, Spain; 10 Institute of Biomedicine (IBIOMED), University of León, 24071 León, Spain

**Keywords:** PBMC, neutrophils, macrophages, prednisolone, methotrexate, leflunomide, biofilm, chemokines, *Mycobacterium abscessus*

## Abstract

Immunosuppressive therapies increase the risk of infection, but there is little information regarding their effects on cellular antimycobacterial activity. In this context, the aim was to evaluate in vitro the impact of commonly used immunosuppressive drugs on the ability of peripheral blood mononuclear cells (PBMCs), neutrophils (polymorphonuclear cells, PMNs), and monocyte-derived macrophages (MDMs) to control *Mycobacterium abscessus*. Biofilm formation was assessed by quantifying bacterial colonies in cellular cultures (BCCCs) and bacterial viability by colony-forming units (CFUs). BCCCs showed significant differences among treatment conditions in PBMCs. The median (interquartile range) BCCC values for tacrolimus (TAC) 16.5 (41), everolimus (EVE) 11 (33), methotrexate (MTX) 12.5 (22) and leflunomide (LEF) 11 (29) were all significantly higher than the negative control (DMSO) 5 (14), indicating that these immunosuppressants impaired the ability of PBMCs to restrict BCCC formation. Log-transformed CFUs also varied across treatments in PMNs. Mycophenolic acid (MPA) 5.98 (2.61) and EVE 5.85 (2.77) increased LogCFU recovery compared with DMSO 5.58 (2.63), whereas MTX 5.18 (2.74) decreased it. In contrast, immunosuppressants had no significant overall effect in MDM cultures. Interestingly, 6-mercaptopurine (6MP) affected the size of colonies. Prednisolone, as expected, but also MTX and LEF, inhibited the expression in infected PBMCs of IL-1β, IL-1Ra, IL-6, CCL3, CCL5, CXCL8 and TIMP-2, whereas IL-10, CCL2 and CXCL7 expression remained essentially unchanged. Unexpectedly, methotrexate promoted CXCL8 expression, a chemokine for PMNs. These results show that commonly used immunosuppressive drugs can differentially modulate the antimycobacterial activity of PBMCs and innate immune cells, affecting both mycobacterial viability and biofilm formation.

## 1. Introduction

Immunosuppressive therapies are widely used in the management of autoimmune and inflammatory diseases, as well as in the prevention of organ transplant rejection and graft-versus-host disease. Immunosuppressive drugs include glucocorticoids, calcineurin inhibitors, mTOR inhibitors and antimetabolites that interfere with nucleic acid synthesis [1]. Glucocorticoids such as prednisolone (PSL) have strong anti-inflammatory and, at high doses, immunosuppressive activities, affecting T cell activation and antibody production [2]. The other drugs have different mechanisms of action, interesting for their effect on the adaptive immunity. The immunophilin-binding drugs are calcineurin inhibitors (tacrolimus, TAC) [3] or target-of-rapamycin inhibitors (everolimus, EVE) [4]. TAC affects the phosphatase activity of calcineurin in T cells, inhibiting the activation of the nuclear factor of activated T cells (NFATs) and the transcription of the gene encoding IL-2, whereas the immunosuppression activity of EVE is exerted through blocking the cell division of activated T cells. The inhibitors of nucleoside synthesis (methotrexate, MTX) [5,6] are further divided between purine synthesis inhibitors (mycophenolic acid, MPA, 6-mercaptopurine, 6MP, or its prodrug azathioprine) [3] and pyrimidine synthesis inhibitors (leflunomide, LEF) [7]. These inhibitors limit DNA synthesis and consequently cellular proliferation, in particular, the expansion of T and B cells. Nevertheless, it was soon discovered that these broad-acting molecules also influenced innate immunity. Macrophage and neutrophil (polymorphonuclear cells, PMNs) cytokine production, receptor surface expression, modulation of adhesion molecules, apoptosis, chemotaxis, phagocytosis or reactive oxygen species production, among other functions, are modulated by glucocorticoids and small-molecule immunosuppressants [8,9]. Additionally, because cytokines and chemokines are important regulators of cellular recruitment and antimicrobial activity, their modulation may influence host control of infections.

Despite having reasonably good safety profiles, some adverse effects have been described for these molecules, such as an increased risk of infection, as the most frequent, but also malignancy and metabolic diseases [10,11]. The immunosuppressive molecules most strongly connected to increased susceptibility to bacterial infection are the glucocorticoids, depending on dose, duration and intensity [2]. MTX has been linked to infection in rheumatoid arthritis but not in other inflammatory rheumatic diseases [12]. Quite the opposite, there is evidence that MTX may play a protective role against infection in early inflammatory arthritis [13]. MPA is not considered to increase the risk of bacterial infection [14], but occasional associations have been reported [15]. Azathioprine/6MP has also been connected to bacterial infections [16,17]. Similarly, LEF has been significantly associated with pneumonia [18]. TAC has been identified as a risk factor for tuberculosis or involved in other infections [19,20]. Finally, EVE has also been related to an increased risk of infection [21]. Nevertheless, it is important to note that the summarized publications focus solely on studies where infection risk could be linked to a single immunosuppressive therapy. Most reports of infections, however, describe patients receiving multiple immunosuppressive agents simultaneously, often with synergistic effects that may amplify the risk of infection.

To study the increased susceptibility to infection in immunosuppressed patients, mycobacteria provide an especially pertinent model organism. Besides the clinical importance of tuberculosis [22], infections by nontuberculous mycobacteria (mycobacteria other than *Mycobacterium tuberculosis* complex and *M. leprae*) are a progressively recognised global health issue [23]. In particular, *M. abscessus* has emerged as an important opportunistic pathogen, especially in immunocompromised individuals, who are associated with difficult-to-treat infections due to its intrinsic antibiotic resistance [24]. Although *M. avium* complex remains the most frequently isolated NTM in pulmonary disease, ranging from 9 to 58% in Africa to 51 to 85% in North America, *M. abscessus* complex is increasingly reported, with prevalences of 3–18% in North America and 7–45% across Asia [25]. The main objective of this study was to evaluate the influence of different immunosuppressants on the in vitro antimycobacterial activity of several cellular models, namely peripheral blood mononuclear cells (PBMCs), monocyte-derived macrophages (MDMs), and PMNs, using *M. abscessus* as the experimental model. In addition, we examined differences in cytokine expression in infected cells exposed to the immunosuppressants and assessed their correlation with BCCC formation. After three days of infection, distinct outcomes were observed depending on the cellular model and the parameter analyzed: either biofilm formation, quantified as bacterial colonies in cellular cultures (BCCCs), or bacterial survival, measured as colony-forming units (CFUs).

## 2. Results

### 2.1. Cellular Antimicrobial Activity Against M. abscessus

**Figure 1:**
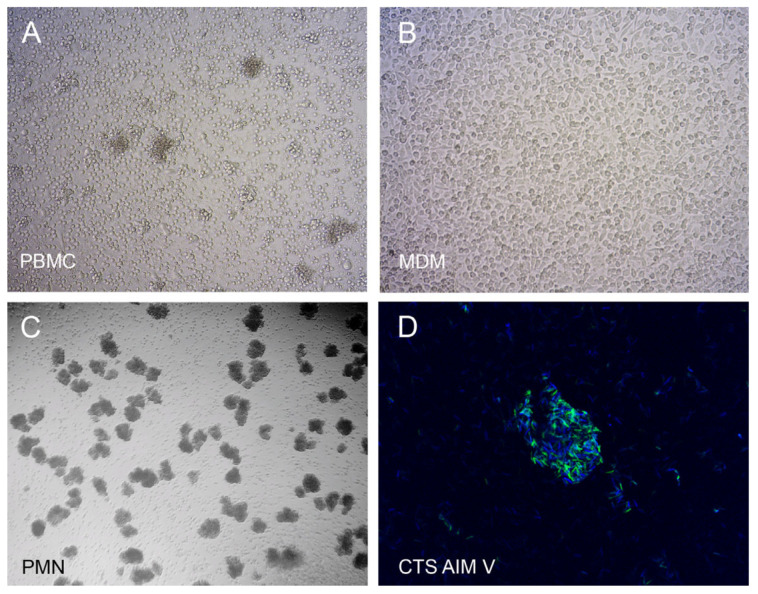
Biofilm characterization. Cells (2 × 10^5^) were infected with 200 *M. abscessus* bacilli and incubated for three days. (**A**) PBMCs. (**B**) MDMs. (**C**) PMNs. (**D**) *M. abscessus* colony grown for four days in serum-free medium CTS AIM V and stained with fluorescein isothiocyanate (protein, green fluorescence) and calcofluor white (β-polysaccharides, blue fluorescence). Microphotographs were taken at 10× (phase contrast, **A**–**C**) or 63× (confocal, **D**) magnifications.

PBMCs, PMNs or MDMs were infected for three days with a low dose of *M. abscessus* (~200 bacteria). After enumeration of BCCCs, cells were lysed to release the intracellular bacteria and inoculated in mycobacterial medium to quantify CFUs. Figure 1 shows the development of BCCCs in PBMCs (Figure 1A), MDMs (Figure 1B), and PMNs (Figure 1C) cultures, as well as the biofilm staining of an *M. abscessus* colony (Figure 1D).

#### 2.1.1. Biofilm Formation

**Figure 2:**
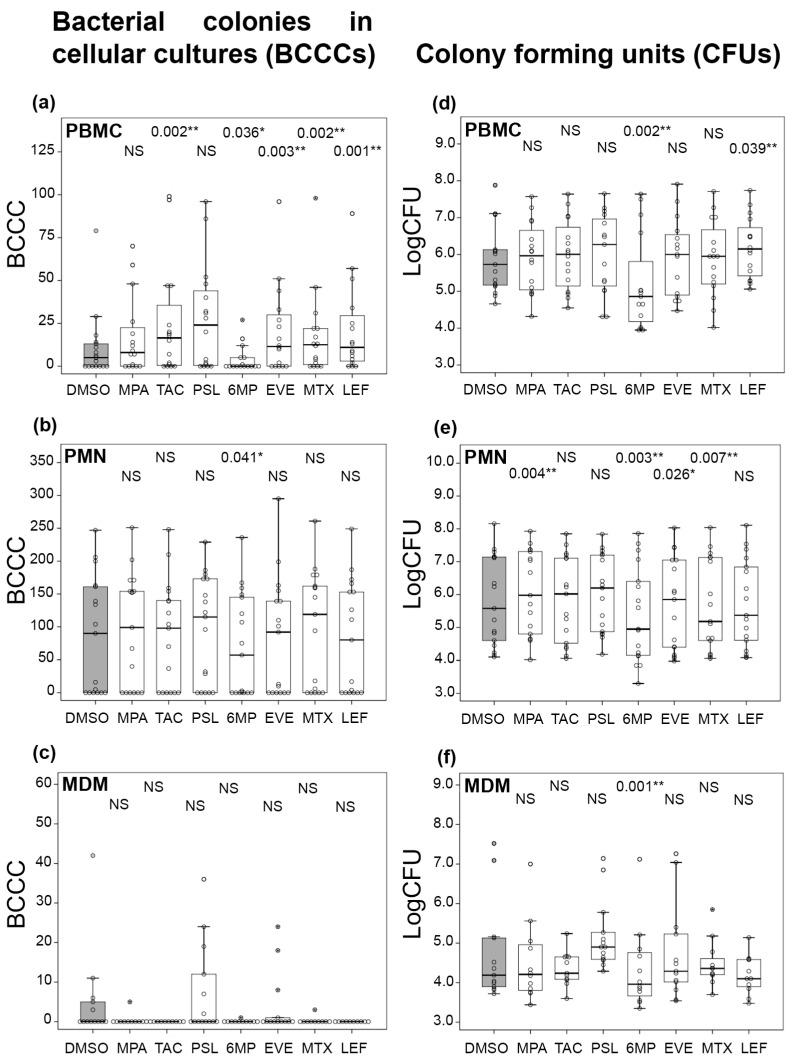
Mycobacterial growth in immunosuppressed cells. Cells (2 × 10^5^) were infected with 200 *M. abscessus* bacilli and incubated for three days before enumerating BCCCs (**a**–**c**). Cells were then lysed, supernatants diluted in mycobacterial 7H9 medium, and incubated for 2–3 days to allow formation and enumeration of CFUs (**d**–**f**). Infected cells were PBMCs (**a**,**d**), PMNs (**b**,**e**) and MDMs (**c**,**f**). Cells were exposed to the immunosuppressants mycophenolic acid (MPA), prednisolone (PSL), 6-mercaptopurine (6MP), methotrexate (MTX), leflunomide (LEF), tacrolimus (TAC) and everolimus (EVE). The vehicle in which immunosuppressants were diluted (DMSO) was the negative control (grey box). Data are represented as box plots, and the Wilcoxon test was used to compare the number of BCCCs or CFUs in each immunosuppressant with the negative control (n = 17). CFU data were logarithm transformed (LogCFU) for easier visualization. Star-marked circles denote outliers. * *p* < 0.05 and ** *p* < 0.01 were considered significant. NS: Not significant.

The number of BCCCs formed was always below 100 in PBMCs (Figure 2a), but it could be even more than the inoculated number of bacteria in PMNs (Figure 2b). However, most of the monocyte samples did not allow the growth of any BCCCs (Figure 2c). In comparison with the negative controls (dimethyl sulfoxide, DMSO), no statistically significant differences were observed in the effect of immunosuppressants in either PMNs or MDMs, with the exception of 6MP in PMNs, in which the number of BCCCs was lower than in DMSO. In contrast, TAC, EVE, MTX and LEF interfered with the PBMC inhibitory activity exhibited in DMSO, with the growth of a larger number of BCCCs. In this cellular model, neither MPA nor PSL showed significant differences with DMSO. Similar to the PMN model, in 6MP, there were significantly fewer BCCCs than in DMSO. In all cell types, there were always samples in which no BCCCs were formed, suggesting that some cells were especially resistant to biofilm formation.

#### 2.1.2. Mycobacterial Survival

There is not a perfect correlation between BCCCs and CFUs because the size of colonies varied, with larger colonies containing more bacteria, and mycobacterial multiplication did not always translate into colony development. For this reason, no significant differences with DMSO were observed for CFUs in PBMCs (Figure 2d), but notable changes were apparent in PMNs (Figure 2e). LEF was the only immunosuppressant with a significantly larger number of both BCCCs and CFUs in PBMCs. In contrast to BCCCs, CFU counts in PMNs were significantly larger for MPA and EVE as compared with DMSO. Interestingly, the MTX median was smaller than that of DMSO. The pattern displayed by MDMs was comparable for both BCCCs and CFUs, with no general differences with DMSO (Figure 2f). The number of CFUs in MDMs was an order of magnitude lower than in PBMCs or PMNs.

Two particularly intriguing immunosuppressive molecules were 6MP and PSL. The size of mycobacterial colonies was remarkably smaller in the 6MP treatment. For this reason, some BCCCs never formed, and the number of mycobacteria per colony was much lower. As a consequence, all the medians were always below that of the DMSO treatment and were not significant only for BCCCs in MDMs, likely because the number in DMSO was also very low. Regarding PSL, it was surprising that no significant differences were detected in any of the cellular models or molecules, provided strong immunosuppressive activity was observed in clinical settings.

### 2.2. Cytokine Expression in Infected PBMCs

**Figure 3:**
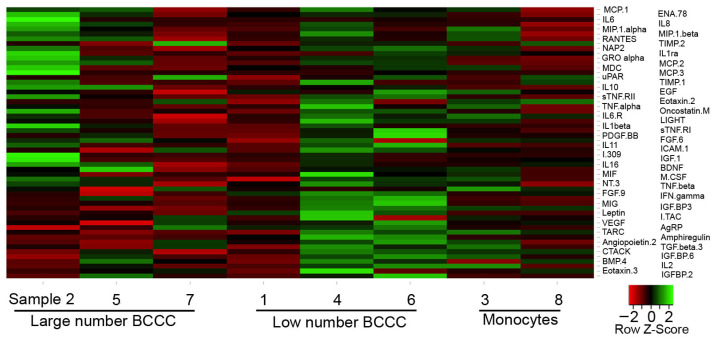
Cytokine expression in infected cells. PBMCs (5 × 10^5^) were infected with *M. abscessus* (5 × 10^5^) and incubated overnight. Cytokine array membranes were incubated with supernatants, and chemiluminescent signal was registered in digital counts units. Analyzed supernatants were obtained from infections in which a large number (samples 2, 5 and 7) or a low number (samples 1, 4 and 6) of BCCCs were formed. For comparison purposes, supernatants of MDMs infected under the same conditions were also analyzed (samples 3 and 8). For the elaboration of the heatmap, the cytokines in sample 2 were ordered from highest to lowest intensities, and compared by row with the intensity of the cytokines in the other samples. Higher and lower expressions were colored green and red, respectively.

To analyze the modulation of cytokine expression by immunosuppressants and its influence on BCCC formation, we first compared the pattern of expression between PBMCs that allowed the formation of a large number and a low number of BCCCs. We selected supernatants from infections in which the formation of BCCCs were the three highest (>22 BCCCs, samples 2, 5 and 7) and the three lowest (<8 BCCCs, samples 1, 4 and 6), and studied the expressed cytokines using a protein array designed for the detection of 120 cytokines. Cytokines with less than three digital count units in all the samples were deemed non-expressed. Out of the 120 cytokines, 56 were considered to have a detectable level of expression (Figure 3). In the group of large numbers of BCCC samples, 5 and 7 had a similar pattern of expression, but different from that of sample 2. In the low number of BCCC group samples, samples 4 and 6 were comparable but different from sample 1. MDM patterns from samples 3 and 8 were also quite different between them. These results show a high degree of heterogeneity between samples, with no clear correlation between the expression of particular cytokines and the number of BCCCs formed. The 10 molecules with the highest intensity among the eight samples were MCP-1/CCL2, IL-8/CXCL8, MIP-1β/CCL4, TIMP-2, NAP-2/CXCL7, MIP-1α/CCL3, RANTES/CCL5, IL1-Ra, uPAR, and ENA-78/CXCL5.

### 2.3. Influence of Immunosuppressants on Cytokine Expression

**Figure 4:**
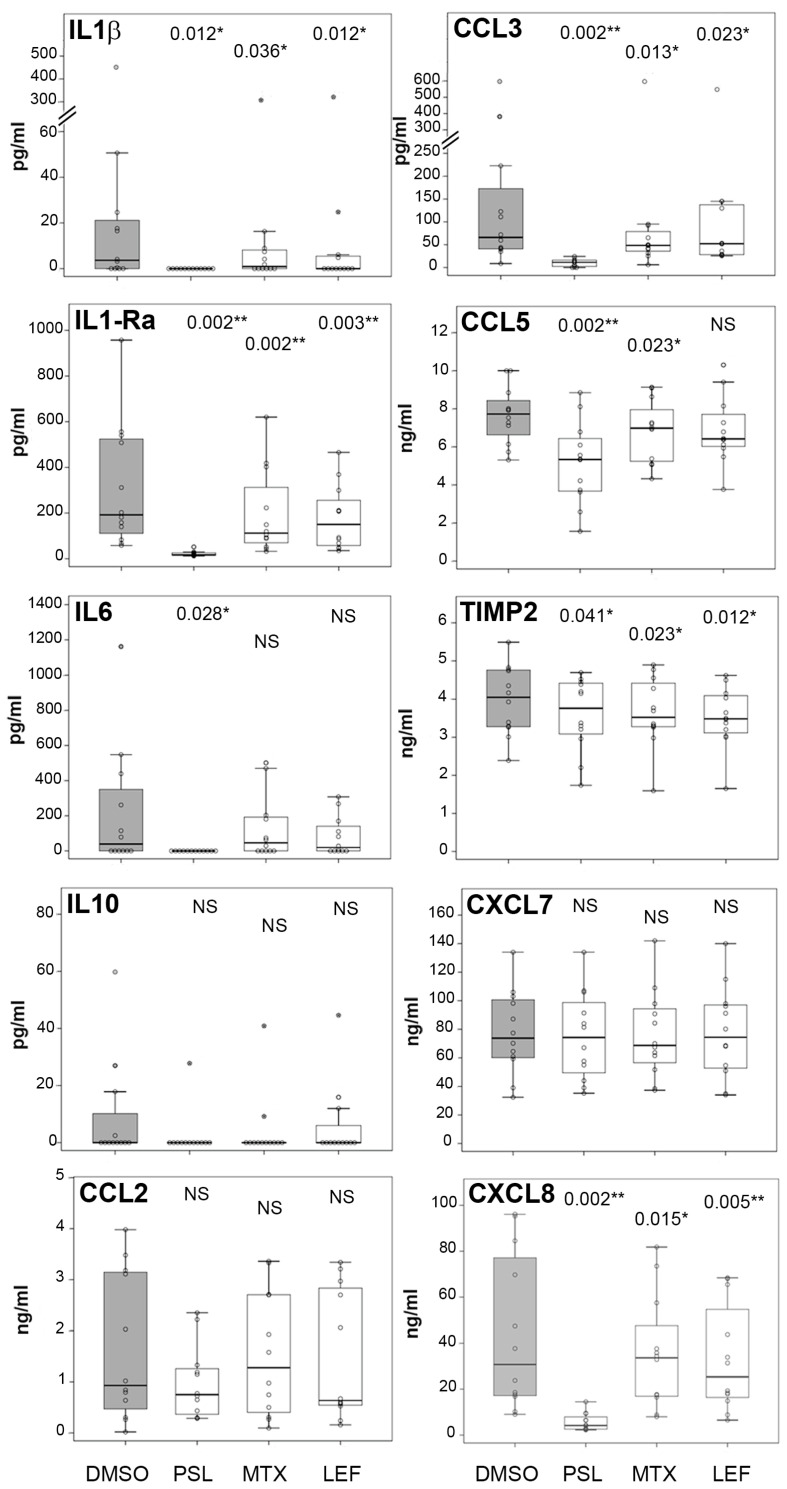
Influence of immunosuppressants on cytokine expression. PBMCs (2 × 10^5^) were infected with *M. abscessus* (2 × 10^5^) in the presence of prednisolone (PSL), methotrexate (MTX) or leflunomide (LEF) and incubated overnight. Cytokines were quantified by ELISA. The vehicle in which immunosuppressants were diluted (DMSO) was the negative control (grey box). Data are represented as box plots, and the Wilcoxon test was used to compare the concentration of the indicated cytokine in each immunosuppressant with the negative control (n = 12). Star-marked circles denote outliers. * *p* < 0.05 and ** *p* < 0.01 were considered significant. NS: Not significant.

From the protein array experiment, we manually selected 10 cytokines among the most expressed and others that we expected to be functionally relevant. Two of them were pro-inflammatory (IL-1β and IL-6), two anti-inflammatory (IL-1Ra and IL-10), three CCL chemokines (CCL2, CCL3 and CCL5), two CXCL chemokines (CXCL7 and CXCL8), and one inhibitor of metalloproteinases (TIMP-2). We studied by ELISA their expression in PBMC infected cells exposed to prednisolone, methotrexate or leflunomide and observed a variable pattern of production depending on the cytokine and the immunosuppressant (Figure 4).

No significant differences between the negative control DMSO and the immunosuppressants were found for IL-10, CCL2 and CXCL7. As would be expected from the powerful anti-inflammatory ability of PSL, it was the molecule with the highest inhibitory capacity, with the only exception of TIMP2, which was less expressed in both MTX and LEF. The comparative inhibition exerted by MTX and LEF varied with the cytokines. It was stronger for MTX in IL-1Ra and for LEF in CXCL8. The degree of inhibition was analogous for IL-1β, CCL3 and TIMP2. Inhibition by LEF was not significant for IL-6 and CCL5, and by MTX for IL-6.

### 2.4. Influence of Cytokines on BCCC Formation

**Figure 5:**
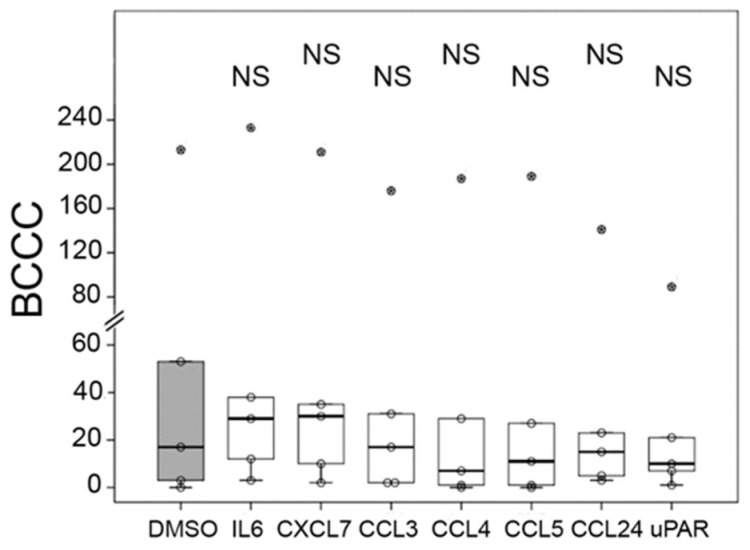
Influence of cytokines in the formation of BCCCs. PBMCs (2 × 10^5^) were infected with 200 *M. abscessus* bacilli and incubated with the indicated cytokines (25 ng/mL) for three days before enumerating BCCCs. The vehicle in which the cytokines were diluted (DMSO) was the negative control (grey box). Data are represented as box plots, and the Wilcoxon test was used to compare the number of BCCCs in each cytokine with the negative control (n = 5). Circles with a star inside are outliers. NS: Not significant.

To analyze the potential inhibitory capacity of cytokines on BCCC formation, we added to infected PBMCs several of the cytokines expressed in the array experiment, the chemokines CXCL7, CCL3, CCL4, CCL5 and CCL24; the proinflammatory IL-6; and the biomarker of inflammatory diseases, urokinase plasminogen activator receptor (uPAR). Nevertheless, no significant differences in the number of BCCCs with the negative control (DMSO) were observed (Figure 5).

## 3. Discussion

The main clinical benefit of these immunosuppressants is the downregulation of adaptive immunity, classically considered the main reason for the increase in infection risk [26]. Nevertheless, the inhibition of innate immunity has long been acknowledged to play a role as well [8]. We have investigated the influence of immunosuppressants on cellular antimycobacterial activity, analyzing the ability of PBMCs and innate immune cells (PMNs and MDMs) to restrict biofilm formation and mycobacterial survival and multiplication. We have addressed the study of biofilm through the enumeration of BCCCs because aggregates, such as mycobacterial colonies, have been recognized as a form of biofilm that is not attached to a surface [27]. The cellular capacity to hinder biofilm formation is appreciated by comparing PBMCs and PMNs. Despite a comparable number of CFUs in both cellular models, the number of BCCCs is more than double in PMNs, suggesting that PBMCs inhibit biofilm formation without affecting mycobacterial viability. The study of biofilm has clinical implications because its importance is increasingly perceived in mycobacterial infections [28].

Immunosuppressants have been found to have a direct impact on mycobacterial viability. Rapamycin (functionally analogous to EVE), TAC [29], MTX and 6MP (derived from the prodrug azathioprine) [30] inhibit the growth of *M. avium* and *M. avium* subsp *paratuberculosis*. The degree of inhibition depends on the species, the strain and the immunosuppressant dose. At the dose used in our study (≤5 μg/mL), we did not observe any inhibition of the 296aba *M. abscessus* strain by the immunosuppressants, with the exception of 6MP, which caused a markedly reduced colony size. The reason for this observation is likely related to the ability of 6MP to interfere with the mycobacterial biosynthesis of cyclic-di-GMP [31], a molecule involved in quorum sensing and biofilm formation [32]. The consequence was that 6MP was always the molecule that resulted in the lowest number of either BCCCs or CFUs, regardless of cellular activity. It should be stressed that azathioprine did not have the same effect.

Macrophages are the main cellular target of intracellular mycobacteria [33], and they may be suitably studied because monocytes differentiate in vitro to macrophages (monocyte-derived macrophages). Our results showed that the capacity of mycobacteria to multiply intracellularly or form extracellular biofilm was hampered, and immunosuppressants had no significant effect on any of those biological outcomes. In contrast, the number of BCCCs in PMNs was large, indicating that they could not limit biofilm formation. This result was not surprising because PMNs are viable in vitro for only a few hours [34], although they may exhibit bacteriostatic activity while they are viable. This early PMN activity explains the differences observed; however, it may not accurately reflect the biological dynamics of PMN accumulation at the site of infection, where newly recruited PMNs continuously arrive. The differences detected are therefore likely due to extracellular bacterial replication occurring after the spontaneous apoptosis of PMNs. Mycobacterial growth (CFU) was higher in the presence of both MPA and EVE. This finding may have clinical relevance, as PMNs are the first cells recruited to the site of infection [35], and these immunosuppressants may hinder effective early control of mycobacterial proliferation. The role of PMNs in these infections is controversial [34], but we have previously proposed that in the initial stages of infection, an appropriate neutrophilic response may prevent biofilm formation [36]. Besides 6MP, the only immunosuppressant that induced a significantly lower number of CFUs was MTX in PMNs.

The more intense immunosuppressive effect was observed for BCCC formation in PBMCs by TAC, EVE, MTX and LEF. All of them hindered the ability of PBMCs to restrict biofilm formation. Considering that the cellular compositions of PBMCs are lymphocytes and monocytes, we do not know the reason for the dramatic differences in BCCC formation between PBMCs and MDMs. It would seem that the number of monocytes in PBMC (approximately 10^4^–6 × 10^4^ in 2 × 10^5^ PBMCs) is enough to phagocytose the mycobacteria (200 bacilli), but inhibition of the lymphocyte fraction may be involved in the observed effect. The only molecule that exhibited immunosuppressive activity in both BCCCs and CFUs was LEF, an in vitro result with unknown relevance in the clinical setting. Our results align with the lack of influence on cellular antimycobacterial activity reported for EVE [37]. The observation that PSL did not enhance bacterial multiplication or biofilm formation in any cellular model was unexpected, given its potent immunosuppressive activity, as reflected by the marked reduction in cytokine expression. Nevertheless, the median of PSL was always larger than that of any other treatment, with the exception of BCCCs in PMNs with MTX, which were nearly the same. Additionally, the dispersion of the data was usually larger than in the other treatments. Notably, dexamethasone, another widely used glucocorticoid, inhibits the multiplication of *M. tuberculosis* in MDMs [38].

The analysis of immunosuppressants in cytokine expression of infected cells showed that, as anticipated, PSL markedly suppressed the production of most cytokines. The only deviations were IL-10, CCL2 and CXCL7. The influence of PSL on cytokine expression varies depending on the cellular model and the stimulus. Inhibition of IL-10 production by methylprednisolone has been described in activated PBMCs [39], but the expression was increased in non-activated PBMCs [40]. CCL2 is also downregulated by prednisolone in activated PBMCs from healthy controls but not from Wegener’s granulomatosis patients [41]. Nevertheless, other monocyte chemokines (CCL3 and CCL5) were inhibited by PSL. An analogous trend was observed with PMN chemokines. While CXCL8 was strongly inhibited, CXCL7 was not affected. Given the apparently redundant roles of these chemokines, the biological implications of these findings are uncertain. The rest of the cytokines with significant differences in their expression were inhibited by MTX and LEF, with the exception of CXCL8 in MTX, which was significantly more expressed than in the control. It is striking that a chemokine for PMNs is more expressed, and the number of CFUs in PMNs is lower than in the control under the influence of MTX. These results are not easy to explain, but may be related to the protective role MTX exhibited in early inflammatory arthritis [13].

In this study, no consistent pattern of cytokine expression that correlated with biofilm formation was identified. Despite the analysis of 120 cytokines, none of them was strictly linked to either promotion or inhibition of BCCC formation. Furthermore, the addition of selected cytokines to the cellular infection seemed to play no role in the number of BCCCs. Although it is conceivable that cytokines do not influence the cellular ability to inhibit BCCCs, or that cytokines other than those analyzed here may modulate BCCCs, one possibility that we have not tested yet is that BCCC inhibition may require the concurrent participation of two or more cytokines.

Our work raises several important questions that should be addressed in future studies. First, it will be essential to elucidate how PBMCs inhibit BCCC formation and to determine the factors underlying the differences in CFUs observed in infected PMNs, particularly considering their short in vitro half-life. It will also be important to uncover the mechanism by which 6MP inhibits colony development and to assess whether this activity may have clinical relevance. Although we were unable to identify a cytokine that either promotes or inhibits BCCC formation, defining the cellular mechanisms responsible for this process and explaining why BCCCs readily form in PBMCs but barely in MDMs remains a key objective. In addition, the lack of significant effects of PSL in vitro, the immunosuppressant most clinically associated with opportunistic infections, warrants further investigation. Finally, understanding the specific biological mechanisms by which each immunosuppressant modulates the antimycobacterial activity of innate immune cells will be crucial.

## 4. Materials and Methods

### 4.1. Bacterial Strain

*M. abscessus* subsp *abscessus* 296aba is a strain with smooth morphology, isolated from sputum, cultured and individualized as described elsewhere [33]. Briefly, bacteria were grown on 7H11 medium (HIMEDIA, Mumbai, India) supplemented with 10% ADC (albumin, dextrose and catalase, Becton Dickinson, Franklin Lakes, NJ, USA) and 0.5% glycerol. Bacteria scraped from solid media were individualized by ultrasonication, and aliquots were frozen in 20% glycerol at −80 °C.

### 4.2. Cellular Purification and Infection

Cells were purified and infected as detailed elsewhere [33]. Briefly, PBMCs and PMNs were purified by density gradient sedimentation, Ficoll Paque Plus (GE Healthcare, Chicago, IL, USA) and Polymorphprep (Serumwerk, Bernburg, Germany), respectively. CD14^+^ cells (monocytes) were purified from PBMCs by magnetic cell separation (Miltenyi Biotec, Bergisch Gladbach, Germany). Approximately 200 individualized *M. abscessus* bacilli were added to 2 × 10^5^ cells of each of the cellular models in CTS AIM V serum-free medium (Gibco, Thermo Fisher Scientific, Waltham, MA, USA) at a final volume of 100 μL in 96-well plates. The in vitro infections were treated with 5 μg/mL of the immunosuppressants mycophenolic acid (BLDpharm, Pudong New Area, Shanghai, China), prednisolone (Thermo Fisher Scientific, Waltham, MA, USA), 6-mercaptopurine (LKT laboratories, St. Paul, MN, USA), methotrexate (Acros Organic, Thermo Fisher Scientific, Waltham, MA, USA) and A77 1726 (the leflunomide active metabolite, Enzo Farmingdale, NY, USA), and 20 ng/mL of tacrolimus (Apollo Scientific, Denton, Manchester, UK) and everolimus (Cayman Chemical Company, Ann Arbor, MI, USA). All immunosuppressants were diluted in DMSO, which was used as a negative control at 0.01%, the final concentration in the infections. In some experiments, 25 ng/mL of IL-6, NAP-2/CXCL7, MIP-1α/CCL3, MIP-1β/CCL4, RANTES/CCL5, Eotaxin-2/CCL24 (Peprotech, Thermo Fisher Scientific, Waltham, MA, USA), and uPAR (Assay Genie, Dublin, Ireland) were added. Infected cells were incubated at 37 °C and 5% CO_2_. BCCCs were visible at 3 days and were enumerated under an inverted microscope before lysis of cells by ultrasonication. Lysates were diluted in 7H9 liquid medium (Becton Dickinson), supplemented with ADC and 0.5% glycerol, and incubated at 37 °C. CFUs were enumerated after two days under an inverted microscope. BCCCs were visualized by phase contrast in a DMIL inverted microscope (Leica, Wetzlar, Germany), and microphotographs were taken with a Dino-Eye AM423X camera (Dino Lite, New Taipei City, Taiwan).

### 4.3. Cytokine Antibody Array

PBMCs or monocytes (5 × 10^5^ cells) were infected with the strain 296aba (5 × 10^5^ bacilli) in CTS AIM V serum-free medium at a final volume of 1300 μL (48-well plates) and incubated overnight at 37 °C and 5% CO_2_. Supernatants were centrifuged at 16,000× *g* for 5 min, sterilized by filtration with a 0.22 μm pore size microfilter, and frozen at −80 °C. Membranes from the Human Cytokine Antibody Array C1000 kit (AAH-CYT-1000-8, RayBiotech, Peachtree Corners, GA, USA), designed to detect 120 cytokines, were incubated with selected supernatants following the manufacturer’s instructions. Chemiluminescent signal was acquired with a ChemiDoc XRS system (BioRad, Hercules, CA, USA), and intensity of signals was quantified with the Quantity One 1-D analysis software (v. 4.4.1) in digital counts units. Data were analyzed with the Microsoft Excel-based Analysis Software Tool provided by Raybiotech. The heatmap was generated using the Heatmapper expression tool (http://www.heatmapper.ca/expression/ (accessed on 9 February 2026)), selecting ‘row’ as scale type.

### 4.4. Cytokine ELISA

Cytokines in supernatants from independent samples obtained as described in the cytokine antibody array procedure were measured by ELISA. Kits used to analyze cytokines were Human IL-1β, IL-6 and IL-10 ELISA Sets (Diaclone, Medix Biochemica, Espoo, Finland), Elabscience Uncoated Human IL1-Ra, MCP-1/CCL2, MIP-1α/CCL3, RANTES/CCL5 and TIMP-2 ELISA kits (Elabscience, Houston, TX, USA) and DuoSet ELISA development system NAP-2/CXCL7 and IL-8/CXCL8 (R&D, Biotechne, Minneapolis, MN, USA), following manufacturer’s instructions.

### 4.5. Fluorescence and Laser Confocal Imaging

*M. abscessus* (10^3^ bacilli) were cultured in 400 μL of CTS AIM V medium in 24-well plates for four days. Cultures were collected and centrifuged in a Cytospin (Thermo Scientific, Waltham, MA, USA) at 20× *g* for 10 min at room temperature. Slides were fixed in methanol for 30 min, air-dried, and incubated with fluorescein isothiocyanate for protein labeling (20 µg/mL, Sigma-Aldrich, Merck, Saint Louis, MO, USA; green fluorescence) for 30 min. After three washes with phosphate-buffered saline, slides were stained with a 1:1 solution of calcofluor white M2R to visualize β-polysaccharides (Sigma-Aldrich; blue fluorescence) for an additional 30 min. After three washes with phosphate buffered saline, slides were air-dried and mounted with Vectashield antifade medium (Vector Laboratories, Newark, CA, USA). Results were visualized in a Zeiss LSM 800 confocal laser scanning microscope (Zeiss, Jena, Germany). Images were acquired at magnification 63× and analyzed using ZEN (Zeiss, v. 3.10) software.

### 4.6. Statistical Analysis

Data were not normally distributed (Shapiro–Wilk test). Two group comparisons between the negative control (DMSO) and each of the treatments were performed by the Wilcoxon signed-rank test. The statistical program used was PASW Statistics 18 (IBM). In all statistical analyses, a *p* value < 0.05 was considered significant.

## 5. Conclusions

Analyzed immunosuppressants interfered with the PBMC inhibition of *M. abscessus* biofilm formation. They also negatively affected the bacteriostatic activity of PMNs. The expression in infected PBMCs of IL-1β, IL-1Ra, IL-6, CCL3, CCL5, TIMP-2 and CXCL8 was downregulated by PSL, and some of them by MTX and LEF. The main exceptions to these observations were some effects of MTX in PMNs. It enhanced the antimicrobial activity of PMNs and increased the production of CXCL8, a chemokine for PMNs. No correlation was found between the expression of the analyzed cytokines and biofilm formation. Overall, these results support the idea that the impact of immunosuppressive therapy on susceptibility to mycobacterial infection partially depends on the specific drug and the affected innate immune cell.

## Data Availability

The original contributions presented in this study are included in the article. Further inquiries can be directed to the corresponding author.

## Abbreviations

The following abbreviations are used in this manuscript:

BCCCs	Bacterial colonies in cellular cultures
CFUs	Colony-forming units
DMSO	Dimethyl sulfoxide
ELISA	Enzyme-linked immunosorbent assay
EVE	Everolimus
LEF	Leflunomide
MDMs	Monocyte-derived-macrophages
6MP	6-mercaptopurine
MPA	Mycophenolic acid
MTX	Methotrexate
NFATs	Nuclear factor of activated T cells
PBMCs	Peripheral blood mononuclear cells
PMNs	Polymorphonuclear cells
PSL	Prednisolone
TAC	Tacrolimus
uPAR	Urokinase plasminogen activator receptor
